# Destination Memory in Mild Alzheimer’s Disease

**DOI:** 10.3233/BEN-2012-129014

**Published:** 2013

**Authors:** Mohamad El Haj, Virginie Postal, Didier Le Gall, Philippe Allain

**Affiliations:** ^1^Laboratoire de Psychologie EA 2646Université d'AngersAngersFrance; ^2^Laboratoire de PsychologieSanté et Qualité de vieUniversity BordeauxBordeauxFrance; ^3^Centre Mémoire de Ressources et de RechercheCentre Hospitalier UniversitaireAngersFrance

**Keywords:** Alzheimer’s disease, destination memory, inhibition

## Abstract

In order to assess their destination memory, sixteen patients with probable mild Alzheimer Disease (AD), sixteen older adults and 16 young adults were asked to tell facts to pictures. On a subsequent task, they were asked to remember whether they had previously told that fact to that face or not. AD patients showed poorer destination recall than the older adults, and the older adults showed poorer destination recall than the young adults. Our results suggest that destination memory is highly impaired in AD.

## 1. Introduction

Destination memory, a component of episodic memory, can be defined as the ability to remember the destination of produced information (e.g., "did I ask Marie or John to help me with the paper?"). This memory is found to be particularly deteriorated in normal aging [1]. Our main aim was to replicate this finding in Alzheimer's Disease (AD) patients. We also investigated whether this decline is subsumed by executive factors.

## 2. Method

### *2.1. Participants*

Sixteen patients suffering from AD (11 women and 5 men; *Mean* age: 78.39 years, *SD*: 5.71; Mini-Mental State Examination: 22.35, *SD*: 1.48) were recruited from local retirement homes. Their referred neurologists diagnosed probable AD on the basis of the criteria of McKhann and colleagues [2]. Their age and socio-cultural level in terms of years of education were matched with those of a group of 16 older adults (10 women and 6 men; $M$ age: 74.41 years, *SD*: 6.78; Mini-Mental State Examination: 28.86, *SD*: 1.11), respectively, $t(30) = 1.17$, $p > 0.10$, and, $t(30) = 1.26$, $p > 0.10$. The verbal ability of the older adults was matched with that of a cohort of 16 young adults (9 women and 7 men; $M$ age: 20.98 years, *SD*: 3.56), $t(30) = 1.31$, $p > 0.10$.

### *2.2. Materials*

Materials in the destination memory task consisted of 24 familiar facts (e.g., Rome is the capital of Italy) and 24 pictures of celebrity faces (e.g., Elvis Presley). The facts and pictures were reported by an independent sample as being above familiarity level 3 on a l (never encountered) to 5 (very familiar) scale.

In addition to the destination memory task, participants were given executive tasks tapping: shifting, updating, inhibition, spontaneous flexibility and Binding. This procedure has been fully described elsewhere [3, 4]. Table 1 shows the performance on the executive and destination tasks.

### *2.3. Procedures*

In the destination memory task, the experimenter presented a celebrity picture and asked participants to

ISSN 0953-4180/13/$27.50 © 2013 – IOS Press and the authors. All rights reserved

Table 1
Scores obtained on the five executive tasks and the destination measure

| Cognitive function | Task | Young | Older | AD |
|---|---|---|---|---|
| Inhibition | Stroop | 14.31 (3.35)* | 36.06 (11.84)* | 62.47 (11.11) |
| Shifting | Plus-minus | 4.04 (1.99)$^{n/s}$ | 6.07 (4.17)* | 11.09 (8.42) |
| Updating | 2-back | 3.74 (2.89)* | 7.19 (5.68)$^{n/s}$ | 10.35 (5.47) |
| Flexibility | Verbal fluency | 20.01 (5.41)$^{n/s}$ | 22.69 (6.11)* | 16.89 (5.99) |
| Binding | Binding | 0.91 (0.12)$^{n/s}$ | 0.79 (0.28)* | 0.58 (0.32) |
| Destination memory | Destination task | 0.77 (0.19)* | 0.64 (0.28)* | 0.35 (0.29) |

*Note.* Standard deviations given in parentheses; AD = Alzheimer's Disease; the difference between groups was either $^{n/s}$ non-significant or significant at $^*p < 0.05$, as indicated by Post hoc comparisons with Newman-Keuls tests.

tell a fact to that image and so on, until the participants had told each of the 24 facts to a different picture. On a subsequent recognition task, participants were exposed to 20 face-fact pairs: 10 intact and 10 re-paired of previously exposed pairs. They had to determine whether they had previously told that fact to that face or not. A corrected recognition score for each participant was calculated as the proportion of hits (i.e., correct yes responses) minus the proportion of false alarms (i.e., incorrect yes responses) (for a similar procedures, see, El Haj and colleagues [3]).

### 2.4. Results

To test for group differences (young, older adults, and AD patients), a one-way ANOVA was conducted on destination performance. A significant group effect was detected, $F(2, 45) = 17.89$, $p < 0.001$. Post hoc Newman-Keuls tests showed that AD patients performed worse than older adults ($p < 0.05$), and older adults performed worse than young adults ($p < 0.05$). Second, significant correlations were found between destination performance and, among the executive measures, performance on the Stroop, $r = -0.49$, $p < 0.01$, the Plus-Minus, $r = -0.37$, $p < 0.01$, and the Binding, $r = 0.34$, $p < 0.05$, tasks. Third, we conducted a forward stepwise regression with destination performance as the dependent variable and scores on the Stroop, the Plus-Minus and the Binding tasks as predictive variables. These analyses showed that performance on the Stroop task was the main and only variable predicting the destination memory index: 24.3% ($p < 0.001$).

## 3. Discussion

This study produced three findings. First, destination memory was found to be particularly affected in AD patients. Second, significant correlations were detected between destination memory and several executive functions such as inhibition, shifting and binding. Third, among these executive functions, regression analyses suggested inhibition to be a main factor explaining variance in destination memory performance.

Our results extend those of previous studies [1,3] as they show a decline in destination memory in normal aging and even greater decline in AD subjects. The decline in the latter could be due to inhibitory dysfunction. This hypothesis is in line with the theory of Lustig and colleagues [5] who attribute the memory decline observed with normal aging to inhibitory deterioration. Recent reports also relate the memory decline in AD patients to inhibitory dysfunction [3,6].

In conclusion, our findings reflect the impairment that AD patients experience with regard to destination information. They also suggest the involvement of inhibitory processes in this decline.
